# Citronellal perception and transmission by *Anopheles gambiae* s.s. (Diptera: Culicidae) females

**DOI:** 10.1038/s41598-020-75782-3

**Published:** 2020-10-29

**Authors:** Weijian Wu, Shanshan Li, Min Yang, Yongwen Lin, Kaibin Zheng, Komivi Senyo Akutse

**Affiliations:** 1 Institute of Subtropical Agriculture, Fujian Academy of Agriculture Sciences & Zhangzhou Institute of Technology, Zhangzhou, 363001 China; 2 International Centre of Insect Physiology and Ecology, P.O. Box 30772-00100, Nairobi, Kenya

**Keywords:** Entomology, Parasitology

## Abstract

*Anopheles gambiae* s.s. is a key vector of *Plasmodium* parasites. Repellents, which may be a promising alternative to pesticides used to control malaria mosquitoes. Although citronellal is a known mosquito repellent, its repellency characteristics are largely unknown. Determining the specific odorant-binding proteins (OBPs) and odorant receptors (ORs) that detect and transfer the citronellal molecule in *A. gambiae* s.s. will help to define the mode of action of this compound. In this research, we assessed the repellent activity of citronellal in *A. gambiae* s.s. using a Y-tube olfactory meter, screened candidate citronellal-binding OBPs and ORs using reverse molecular docking, clarified the binding properties of predicted proteins for citronellal using fluorescence competition binding assay. Results showed that citronellal had a dosage effect on repelling *A. gambiae* s.s.*.* The 50% repellent rate was determined to be 4.02 nmol. Results of simulated molecular docking showed that the only proteins that bound tightly with citronellal were AgamOBP4 and AgamORC7. Fluorescence competitive binding assays confirmed the simulations. This research determined that citronellal was captured by AgamOBP4 and transmitted to AgamORC7 in *A. gambiae* s.s.. Our study will be beneficial in the further understanding the repellent mechanism of citronellal against *A. gambiae* s.s..

## Introduction

Malaria, which is caused by *Plasmodium* parasites, is the deadliest circumtropical infectious disease^1–4^. Mosquitos in the *Anopheles gambiae* Giles (Diptera: Culicidae) species complex are the important insect vectors of *Plasmodium* parasites^5–8^. The application of chemical pesticides, such as pyrethroids and organophosphates has been the most popular strategy for controlling *A. gambiae* s.s. in recent decades^9–12^. During this time period, pesticide overuse has, unfortunately, led to high levels of insecticide resistance in this mosquito vector, while also causing serious pesticide pollution problems, especially in heavily populated areas^11,13–15^. After becoming aware of the serious problems caused by the buildup of resistance and pollution, many agents have resorted to alternative control measures, including various mosquito-repellent plants such as citronella, lavender and mint to obtain relief from the vector^16–18^. Citronellal is a monoterpenoid that was originally found in the volatile organic compounds (VOCs) emitted from several varieties of mosquito-repellent-plants. It’s reported that citronellal had high evaporation rate in the first 2 h, so as the efficiency of its repellent or attraction always reach the highest level^19^. Although citronellal and its derivatives have been shown to have mosquito-repellent activity against several mosquito species, including *Aedes aegypti* (L.), *A. gambiae* s.s. and *Culex pipiens* L.^20–22^, its repellency efficiency in repelling *A. gambiae* s.s. has not been determined. In addition, the mosquito’s recognition mechanism and response to citronellal is still largely unknown.

Insects detect small volatile molecules (chemical cues) using soluble odorant binding proteins (OBPs)^23–26^, and subsequently transfer the volatile molecules to odorant receptors (ORs) or olfactory ionotropic receptors (IRs)^27–31^. IRs receive chemical signals such as ammonia, lactic acid, and other carboxylic acids^31^, while ORs related with transmission for small molecular, and have a strong possibility to bind citronellal according to a previous study^32^, so we focused on ORs in this study. The ORs or IRs will then activate olfactory receptor neurons located on the same dendrite^33–36^. Obviously, the binding of OBPs to the odorant chemical is the initial step in the reaction of an insect to a chemical cue. To date, approximately 100 known OBPs are known to affect olfaction in *A. gambiae* s.s.. These have been classified into three subfamilies: Classic OBPs, PlusC OBPs and Atypical OBPs^37–40^. Previous studies have discovered more than 80 ORs in *A. gambiae* s.s. as well^41,42^. However, the specific OBPs and ORs that function in detecting and processing citronellal molecules in *A. gambiae* s.s. have yet to be identified.

In this study, we determined the repellent efficiency of citronellal on *A. gambiae* s.s. using a Y-tube olfactory meter, and then predicted which OBPs and ORs were utilized by the mosquito to bind citronellal using the method^43^. In order to establish the underlying mechanism, we then clarified the exact target OBPs and ORs using simulated molecular docking and fluorescence competitive binding tests. It is eager to clear the mechanisms involved in the binding of the ligand (citronellal) and its receptors (OBPs and ORs) will aid in testing new active components for controlling malaria carrying mosquitoes.

## Materials and methods

### Mosquito culture

The *A. gambiae* s.s. culture used in this experiment was origin from Kenya and kept in Institute of Subtropical Agriculture (Fujian Academy of Agriculture Sciences, Zhangzhou, China) for 2 years. Distilled water was provided for *A. gambiae* s.s. oviposition and immature development in an environmental incubator (25 °C, 75% RH, L:D = 12:12) until eclosion of the adults. Larvae were fed on MATSUMO fish food (Japan Matsuno Aquarium Appliance Limited, Akiko District, Kanagawa, Japan). Adults were allowed to mate in net (40 mesh) enclosed cages (40 cm × 40 cm × 40 cm) and fed with 10% litchi honey solution in the first day. Three-day-old, hungry mated adults were placed into a 4 °C refrigerator for 2 min and females were selected for the experiments.

### Dual-choice olfactory test

A glass Y-tube (1.6 cm i.d., 12-cm base , two 8-cm arms at a 45° angle from one another) olfactory meter described in our previous study was used in this experiment^44^. A quantity gradient series (0.01, 0.10, 1, 10 and 100 nmol) of citronellal with the solvent of triethyl citrate (95% and 99% respectively, analytical purity, Sigma-Aldrich LLC., Darmstadt, Germany) was applied to individual cotton balls (5 g), and placed into separate collecting jars (one cotton ball per jar, Figure S1). The control cotton ball with triethyl citrate (without citronellal) was inserted into the other jar. Teflon tube was used to connect all parts of Y-tube olfactory meter. Fresh air was produced by an air pump at a rate of 200 ± 10 ml/min, and purified by active carbon and silicone before flowing into the jars. The headspace of citronellal volatile was combined with the purified air and went through one arm of the Y-tube while purified air without citronellal went through the other arm. The base of the Y-tube was connected to an air collection bottle. One hundred female *A. gambiae* s.s. were individually released in the air collection bottle (2-cm i.d., 8-cm high), where behavioral responses were monitored and recorded over a 300 s period^45^. The collecting jar, Y-tubes, air collection bottle were cleaned with hot water (> 60 °C) after every 10 individuals were tested, and the cotton roll was renewed every 2 h. The olfactory test was repeated five times.

The repellent rate was calculated according to the following formula^46,47^:

$$\left(\mathrm{Repellent\,rate}\right)\mathrm{\%}=\left(1-\frac{\mathrm{mean\,number\,of\,individuals\,selecting\,citronellal}}{100-\mathrm{mean\,number\,of\,individuals\,not\,selecting\,citronellal}}\right)\times 100$$

The repellent rate of 50% individuals (RQ_50_) was estimated based on the quantity of citronellal and the related repellent rate using the Probit analysis model in the SPSS v.20.0 statistical software.

### Citronellal-binding AgamOBPs and AgamOR prediction

We first used reverse molecular docking to predict the reference OBP and OR proteins which can bind with citronellal. In the reverse molecular docking, the 3D structures of citronellal were predicted using Chem3D 17.0 (Thermo Fisher Scientific, Waltham, MA, USA), and submitted to the PharmMapper web service. Reference literature was used to access the citronellal-binding OBPs or ORs^26,48^. Secondly, the amino acid sequences of the OBPs and ORs in *A. gambiae* s.s. (AgamOBPs and AgamORs) was accessed from the protein databases: NCBI, PDB and UniProt. Next, we constructed phylogenetic trees to detect the highly homologous AgamOBPs and AgamORs with the reference proteins by using MEGA7^49^. Finally, the conserved site, hydrophobic domain and protein family of the detected proteins were analyzed using the Cluster Omega and HMMER web services according to their amino acid sequences.

### Auto docking

Auto docking was used to predict the site of the protein (receptor) dock to small molecular (ligand)^43^. The three-dimensional (3D) structures of predicted AgamOBPs and AgamORs were constructed using SWISS-MODEL (https://swissmodel.expasy.org/)^50^, and scored with PROCHECK (https://servicesn.mbi.ucla.edu/PROCHECK/)^51^ and ModBase (https://modbase.compbio.ucsf.edu/). Models of the molecular docking between the receptors and the ligands were visualized in SYBYL-X 2.0 (Rohm and Haas Co., North Andover, MA, USA).

### Fluorescence competitive binding test (FCBT)

For recombinant OBPs, we first need to obtain the OBPs related genes. DNA of *A. gambiae* s.s. female adults was extracted following the procedure used in a previous study^5^. The predicted AgamOBPs and AgamORs were detected using standard PCR with specific respective primers which were designed using Primer Premier 5 (Premier Biosoft, California, USA; Table S1). Each PCR mixture contained 1 μl of *A. gambiae* s.s. cDNA (200 ng·ml^−1^; previously extracted using a DNA extraction kit from Transgen Biotech, Beijing, China), 12.5 μl prime STAR Max Premix (2 ×), 1.5 μl of each primer (10 mM), and 8.5 ml sterilized H_2_O. The PCR cycling conditions were as follows: an initial denaturation at 94 °C for 40 s, followed by 35 cycles at 65 °C for 40 s (denaturing), 59 °C for 50 s (annealing) and 72 °C for 3 min. The PCR products were verified and sent for sequencing. Recombinant proteins were generated by transforming plasmids containing the verified G protein-coupled receptor (GPCR) genes into *Escherichia coli* BL21 (DE3) cells^52^. We then induced recombinant protein expression using 1 mM isopropyl ß-d-1-thiogalactopyranoside at 37 °C for 6 h. The recombinant proteins were purified using two rounds of Ni21 ion affinity chromatography (GE Healthcare, Milwaukee, WI, USA), following the manufacturer's instructions^5,53^; the His-tags were removed simultaneously using recombinant enterokinase (Novagen, Madison, WI, USA), following the manufacturer's instructions^54^. The purified proteins were desalted using extensive dialysis. The size and purity of each desalted protein were determined using 15% sodium dodecylsulphate-polyacrylamide gel electrophoresis (SDS-PAGE)^55^.

The binding affinities between the receptors (both OBPs and OR) and the ligands were tested using FCBT; synthetic citronellal was used as the ligand and four recombinant proteins were used as the receptors. This assay assumes that the receptors are 100% active, and that each receptor can only bind to one ligand^56^.

Recombinant proteins were dissolved in 50 mM Tris–HCl protein buffer to yield a final concentration of 2 mmol l^−1^, respectively. The fluorescence competitive binding were conducted in a 1-cm quartz cell and the fluorescence intensity F95S fluorescence spectrophotometer (Shanghai Lengguang Technology Co., Ltd., Shanghai, China) at 25 °C with the following parameters: excitation of 10 nm; emission slit of 10 nm; sensitivity of 2 s; gain value of 2; excitation wavelength of 337 nm; and an emission wavelength range of 370–500 nm, as a previous study^53^.

First, the binding constant (Kd) for each recombinant protein to the 1-*N*-phenyl-naphthylamine (1-NPN) fluorescent probe was detected. In brief, 2 ml of protein solution and 2 μl of 1-NPN solution were added to the quartz cell and thoroughly mixed for 1 min. Peak fluorescence was continuously recorded until it stabilized and began to decrease. The Kd of the four proteins (receptors) to 1-NPN was then calculated using the Scatchard equation^57^.

Next, the ligands were added to the quartz cell along with each receptor, in order to competitively bind to the fluorescent probe^54^. In brief, 2 ml of protein solution and 2 ml of 1-NPN solution were added to the quartz cell, and the fluorescence peaks were scanned and recorded. Then, 2 μl of each ligand was added and allowed to sit for 1 min before the peak fluorescence was scanned and recorded. We then continued to add 2 μl aliquots of the ligand solution until the fluorescence was less than half of the initial value.

The binding curves were then linearized using a Scatchard plot, which calculates the concentration of the competitor that halves the initial fluorescence intensity (IC_50_). The Kd value for each receptor and ligand was calculated as:

$$\mathrm{Ki}=\frac{{\mathrm{IC}}_{50}}{1+\frac{1-\mathrm{NPN}}{{\mathrm{K}}_{1-\mathrm{NPN}}}}$$

where Ki represented the binding constant, [1 − NPN] represented the free concentration of 1 − NPN, and K_1 − NPN_ was the dissociation constant of each receptor + 1 − NPN complex^58^.

## Results

### Efficiency of citronellal repellency to Anopheles gambiae s.s.

**Figure 1:**
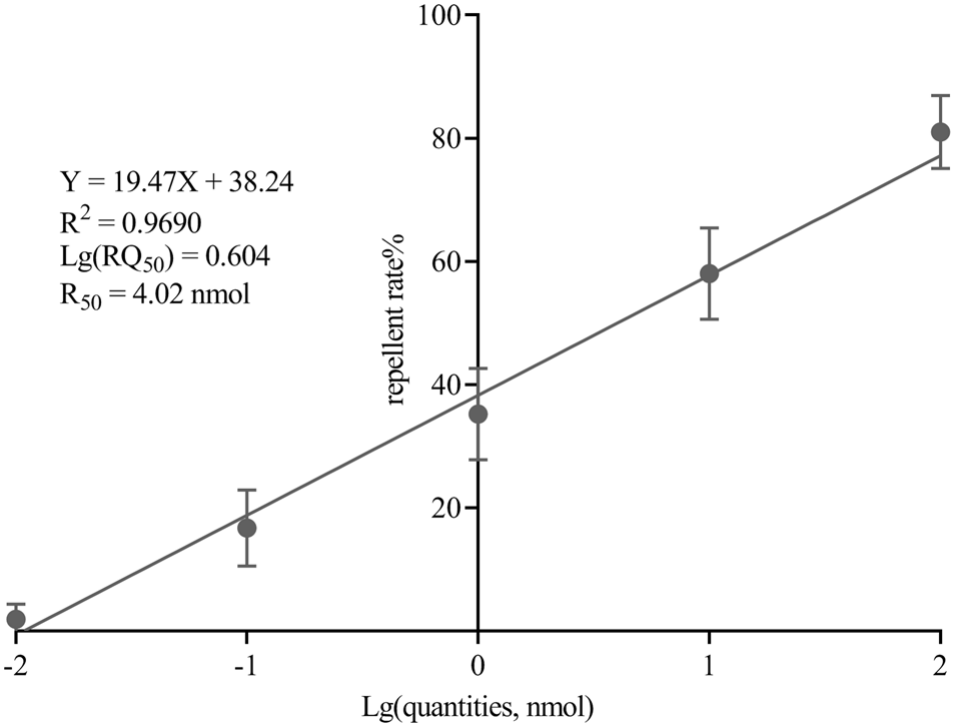
Regression line for citronellal quantity and its related repellant rate. The error bar means mean ± SE.

Results showed that the repellent effect of citronellal on *A. gambiae* s.s. increased in proportion to the quantity at 10^–2^–10^2^ nmol. It means that there was a dose response effect in the citronellal repellency in *A. gambiae* s.s. within the experimental treatments. The repellent rate for 50% of individuals (RQ_50_) was 4.02 nmol (Fig. 1).

### Prediction for citronellal-binding OBPs and ORs in Anopheles gambiae s.s.

**Figure 2:**
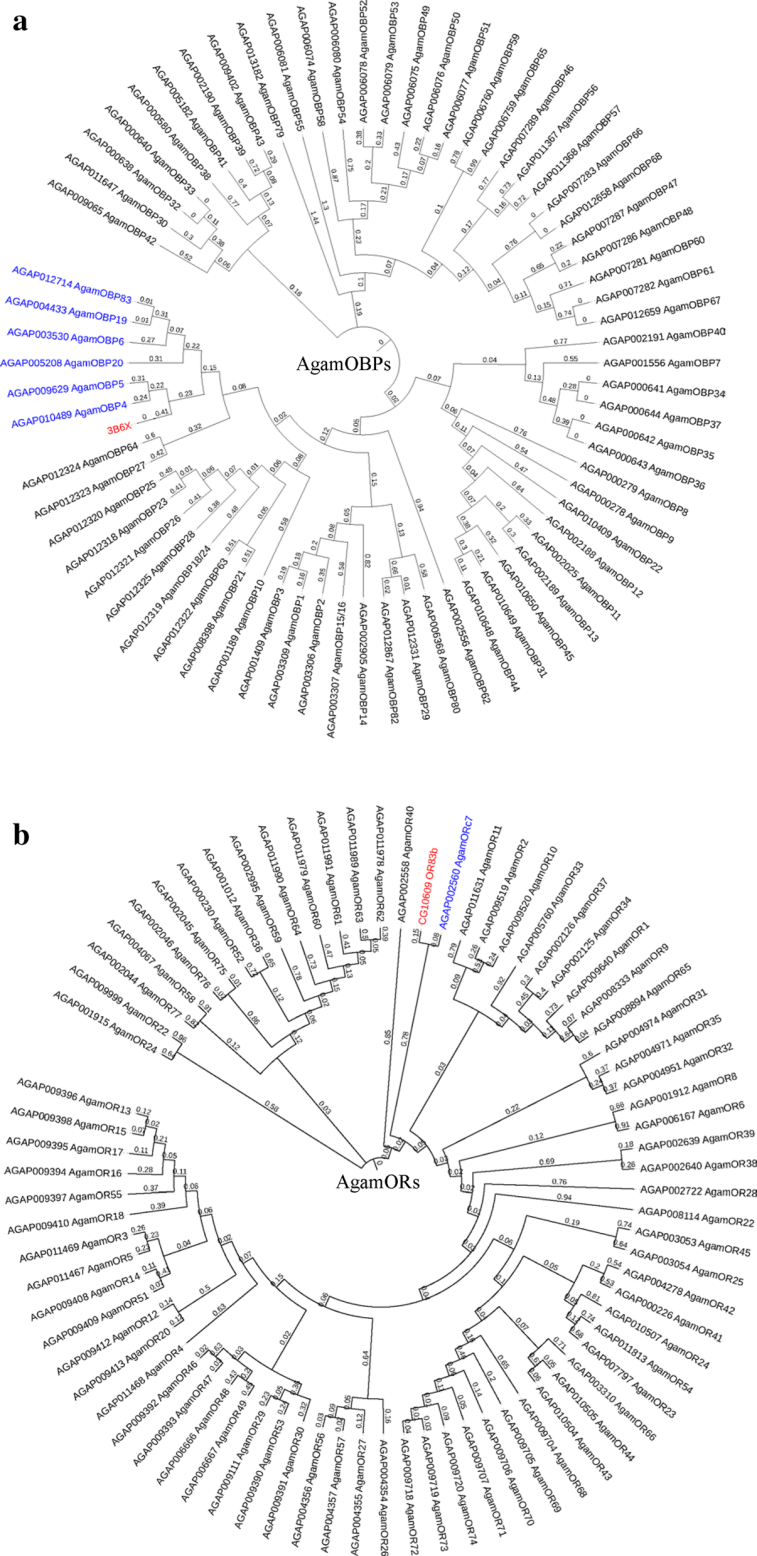
(**a**) Phylogenetic tree for AgamOBPs, and (**b**) AgamORs. Numbers on the branch instead of raw branch length values. Blue color indicated proteins of *A. gambiae* s.s., red color indicated homogenous proteins.

A general odorant-binding protein lush (protein ID: 3B6X) in *Drosophila melanogaster* Meigen (Diptera: Drosophilidae) was predicted as the citronellal-binding OBP but not the OR using PharmMapper Server (Table S2). Amino acid sequences of 70 OBPs in *A. gambiae* s.s. (AgamOBPs) were detected from the protein databases in PBD, NCBI and UniProt. Results showed that amino acid sequences of AgamOBP4, AgamOBP5, AgamOBP6, AgamOBP19, AgamOBP20 and AgamOBP83 were highly homogenous with 3B6X, because these six AgamOBPs clustered into the branch with 3B6X (Fig. 2a). However, an odorant receptor, OR83b (Protein ID: CG10609) in *D. melanogaster* was reported as a citronellal-binding OR in a previous study^59^. Amino acid sequences of 76 ORs in *A. gambiae* s.s. (AgamORs) were detected from the Protein database in PBD, NCBI and UniProt. The results showed that AgamORC7 was highly homogenous with OR83b because it was clustered in the same branch with OR83b (Fig. 2b).

### Prediction and determination of the structure of candidate citronellal-binding AgamOBPs and AgamOR

**Figure 3:**
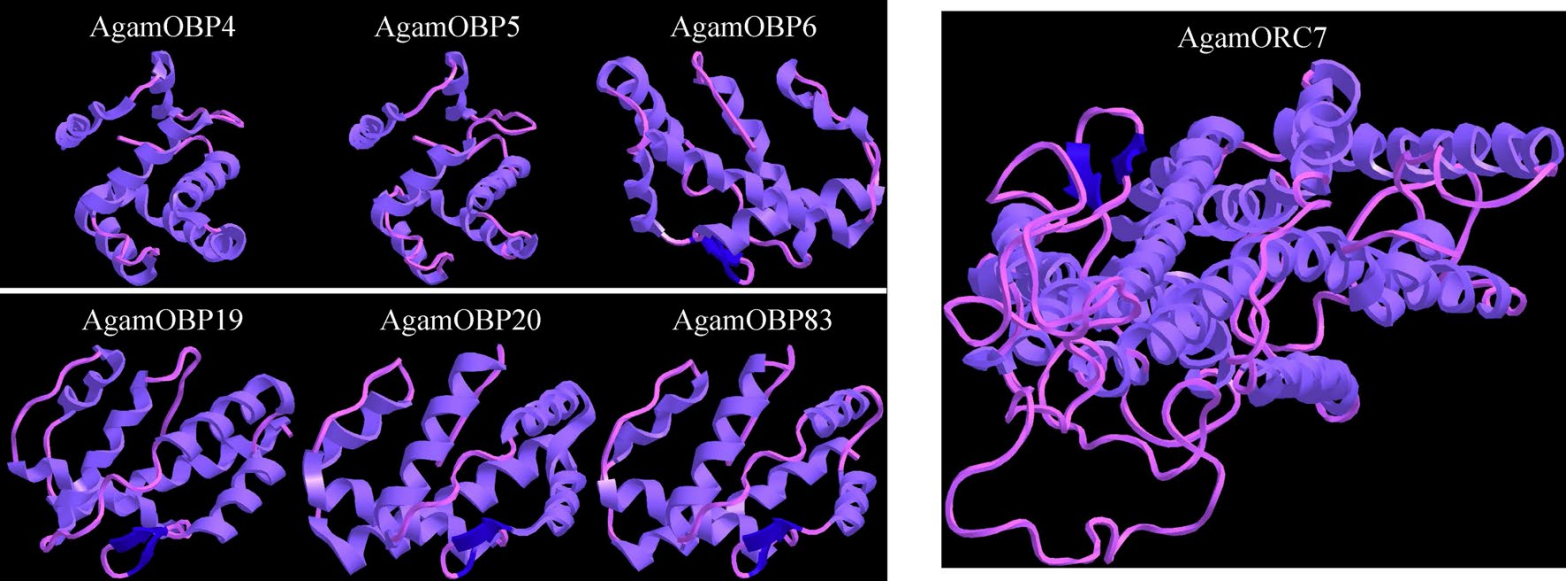
Predicted 3D structure of AgamOBP4, AgamOBP5, AgamOBP6, AgamOBP19, AgamOBP20, AgamOBP83 and AgamORC7.

The amino acid sequences of AgamOBPs and AgamOR were analyzed using the Clustal Omega and HMMER web service. Results of sequence cluster showed that there were 6 cysteine residues and more than 10 hydrophobic binding sites in each of the 6 AgamOBPs amino acid sequences (Figure S2a), meaning that these OBPs belongs to classical OBPs. The hidden Markov models analysis confirmed that these 6 AgamOBPs belong to the PBP-GOBP subfamily (Figure S2b). Results also showed that AgamORC7 like OR83b, has 7 transmembrane domains, and was a typical odorant receptor (Figure S3a and b). We therefore predicted the 3D structure by using SWISS-MODEL and evaluated it using the ModBase and PROCHECKweb service. The 3D structures of the candidate AgamOBPs and AgamOR are shown in Fig. 3. For AgamOBP4 and AgamOBP20, the published 3D structures, which were accessed from the Protein Data Bank in Europe, did not need to be predicted. However, for the others, the 3D structures were reliable according to the value obtained during the evaluation from ModBase (Table S3). By using PROCHECK, all the predicted 3D structures had more than 90% of the residues located in the favored regions (Figure S4), implying that the predicted 3D structures were reliable.

### Simulated molecular docking

**Figure 4:**
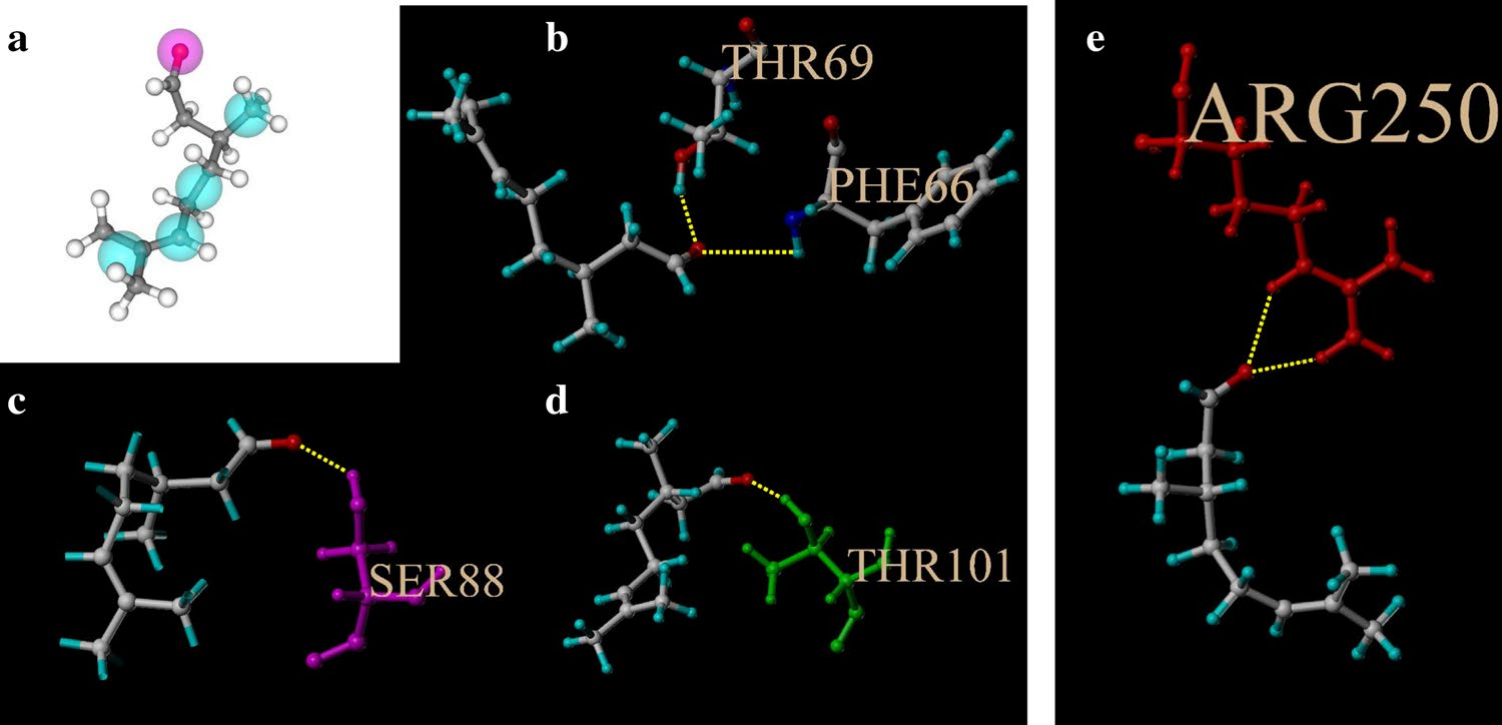
Details of AutoDock for citronellal with predicted proteins. (**a**) possible binding site of citronellal to proteins; (**b**–**e**) binding sites of AgamOBP4, AgamOBP5, AgamOBP20 and AgamORC7 with citronellal. Numbers followed amino means the location of the residues in the amino sequences.

Reverse molecular docking indicated that oxygen atoms on aldehydes of citronellal were the most active docking site (Fig. 4a). Our simulated molecular docking indicated that AgamOBP4, AgamOBP5, AgamOBP20 and AgamORC7 docked with citronellal successfully (Fig. 4b–e), while AgamOBP6, AgamOBP19 and AgamOBP83 could not. For each protein, the total score of auto docking was > 4.5 (Table S4), suggesting that all proteins bound tightly to their ligands. Furthermore, the generated binding sites were observed at PHE66 and THR69 in AgamOBP4, SER88 in AgamOBP5, THR101 in AgamOBP20 and ARG205 in AgamORC7 (Fig. 4b–e).

### Fluorescence competitive binding assay

**Figure 5:**
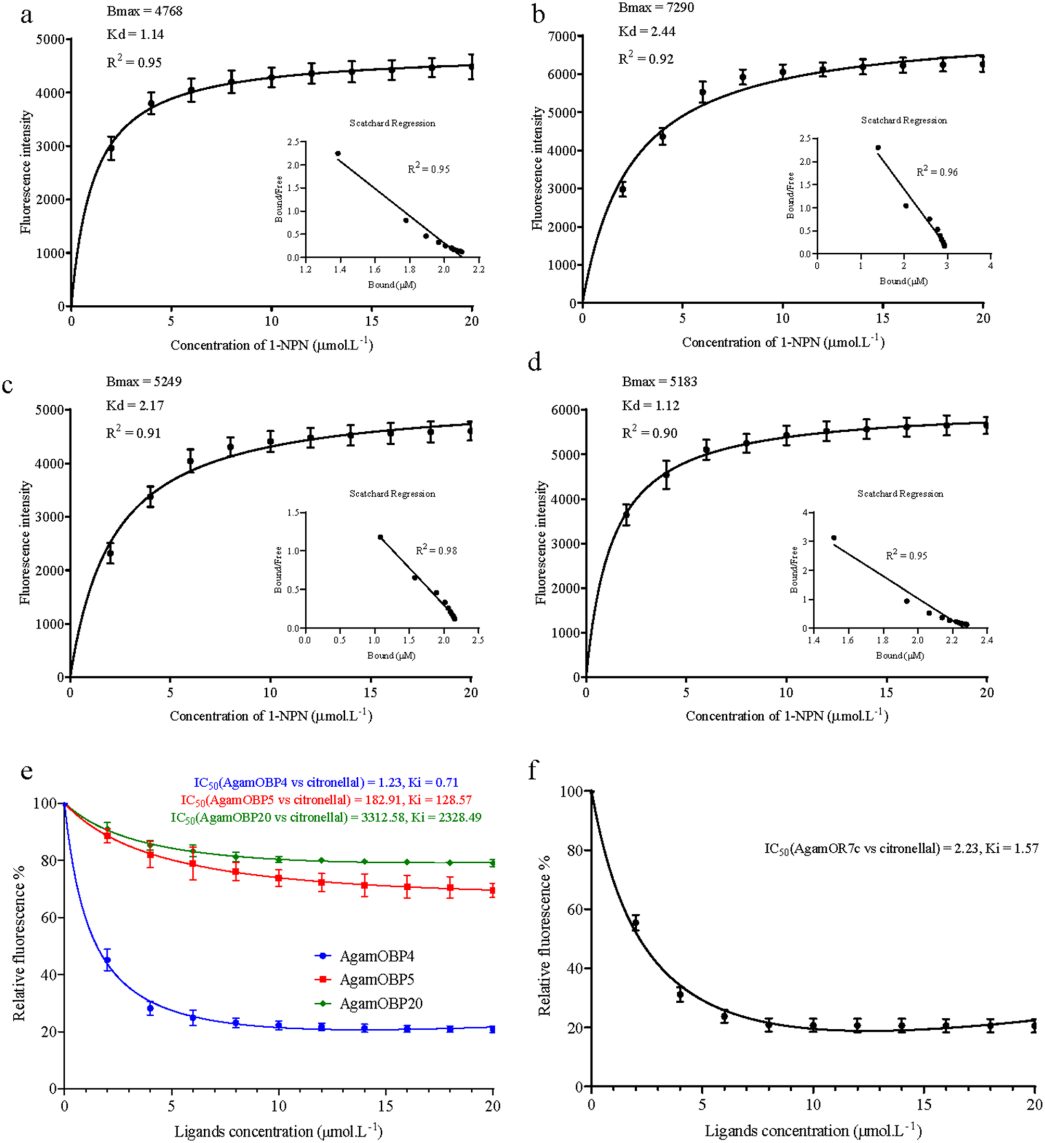
Binding of receptors to ligands. (**a**, **b**, **c**, **e**) Binding curves and relative Scatchard plots for the fluorescent probe (1-NPN) with AgamOBP4, AgamOBP5, AgamOBP20 and AgamORC7; (**d**, **f**) Competitive binding curves for OBPs and OR with citronellal. B_max_, maximal binding capacity; IC_50_, halves the initial fluorescence intensity; Kd, binding constant for each recombinant protein to the 1-NPN fluorescent probe; Ki, Kd value for each receptor and ligand.

Prior to recombination of the target proteins, genes and proteins of the four (AgamOBP4, AgamOBP5, AgamOBP20 and AgamORC7) were detected in gels (Figure S5). Results showed that the binding curves of 1-NPN and each single recombinant protein had good degree of fitting (R^2^ > 0.9), and the relative linearization with showed in Scatchard plot were all fit a straight line (Fig. 5a–d). It means that 1-NPN was a suitable competitive fluorescent reporter for the recombinant proteins. The AgamOBPs competitive binding assays indicated that AgamOBP4 had high binding affinity to citronellal, with an IC_50_ value of 1.23 μM, and Ki value of 0.71 μM. Similarly, AgamORC7 had high binding affinity to citronellal, with an IC_50_ value of 2.23 μM, and Ki value of 1.57 μM (Fig. 5e,f). For AgamOBP5 and AgamOBP20, they could not reduce fluorescent rate down below 50%. This suggests that it is difficult for small molecules to bind to these two proteins.

## Discussion

In this study, we first quantified and tested the repellency of citronellal to *A. gambiae* s.s., and then established the underlying mechanism involved, indicating that *A. gambiae* s.s. bound and transmitted citronellal with odorant binding proteins AgamOBP4 and AgamORC7.

Citronellal gets a lot of attention for its repellency efficiency against mosquito^20,21,32,59,60^, although its exact mode of action is still unknown. This lack of knowledge regarding the mechanism involved in its activity can be attributed to the inconsistency of the diverse methods used to determine the repellency of volatiles in insects^60–68^. In this study, we used a Y-tube olfactory meter which included in its design, most of the critical elements necessary for determining the mosquito-repellent activity of citronellal in vitro. The Y-tube olfactory meter is a widely accepted approach for studying odor detection in insects^69–74^. Our results confirmed that citronellal volatiles repelled *A. gambiae* s.s. and that its efficiency exhibited significant dose response effects in the defined experimental treatments. This signifies that citronellal may be bound by AgamOBPs and transferred to AgamORs by these OBPs.

There are currently more than 70 AgamOBPs and AgamORs in the protein databases (UniProt, PDB database, NCBI). When studying the functions of proteins, much information can be inferred by starting with homologous proteins. By using reverse molecular docking, an OBP, 3B6X, was coupled to citronella in *D. melanogaster*, but no OR was found. Fortunately, a citronellal-docking OR, OR83b, had been found in *D. melanogaster* in a previous study^59^. Therefore, the homologous proteins, 3B6X and OR83b, found in *A. gambiae* s.s., AgamOBP4, AgamOBP5, AgamOBP6, AgamOBP19, AgamOBP20, AgamOBP83 and AgamORC7 were clustered in phylogenetic trees. Classical OBPs have been found to have six conserved cysteine residues and hydrophobic binding sites^75–77^, while ORs which are similar to G-protein coupled receptors have seven transmembrane domains^48,78–80^. Each of these typical traits were found in the amino acid sequences of predicted OBPs and OR, indicating that all of the predicted proteins were eligible.

To analyze the binding details for the predicted proteins and citronellal, the 3D structures for both receptors and ligand were utilized. Because only two 3D structures of predicted protein, AgamOBP4 and AgamOBP20 had been published, the others were predicted by referring to published homologous proteins using SWISS-MODEL. The reliability of the predicted 3D structures was evaluated using PROCHECK. Several other studies on OBPs and ORs have used similar methods and predicted reliable ligands for the receptors^81–85^. We further simulated molecular docking for the predicted protein and citronellal in order to understand their binding traits. Our results showed that only AgamOBP4, AgamOBP5, AgamOBP20 and AgamORC7 can successfully dock with citronellal. According to the AutoDock results, the binding sites in AgamOBP5, AgamOBP20 and AgamORC7 were hydrophilic amino acids, Ser, Thr and Arg respectively, while Phe in AgamOBP4 was found as a hydrophobic amino acid^86–88^. Previous studies have demonstrated that the binding site in OBPs to insoluble odorant molecules should contain a hydrophobic amino acid residue^75,89,90^. This means that, in reality, AgamOBP5 and AgamOBP20 may not bind with citronellal. In addition, there were two 92 Å docking sites on AgamOBP4, implying that AgamOBP4 was capable of binding with two ligands. We also evaluated the binding traits of AgamOBP4, AgamOBP5, AgamOBP20 and AgamORC7 with citronellal through fluorescence competitive binding assay. These results indicated that citronellal can tightly bind to AgamOBP4, but cannot bind well with AgamOBP5 and AgamOBP20. This finding was comparable with the results obtained in AutoDock.

In this study we demonstrated the mosquito-repellent activity of citronellal and quantified the activity with RQ_50_ values. Using our results, one can conjecture that, through direct and indirect evidence, AgamOBP4 solvent in the lymph of mosquito antennae can bind citronellal, and then transmit the information to AgamORC7 on the surface of neuron cells. This research gives a model for exploring the mosquito-repelling mechanism of many other chemicals, e.g. *N*,*N*-diethyl-3-methylbenzamide (DEET). However, we will need a negative control (AgamOBP4 or AgamORC7 mutant *A. gambiae* s.s.) to verify the function of predicted proteins after the genes in the antenna can be knocked down. In addition, citronella oil is among monoterpenoids that produce very high levels of initial spatial repellency followed by relatively lower repellent efficacy, and have a function of intrinsic repellency of their molecules (i.e., their ability to elicit a physiological response at an odorant receptor) and their volatility. It is dictated by their molecular weight, polarity, and the intermolecular forces among the molecules of the repellent compound or with the treated surface (as oppose to sesquiterpenoids), further studies are therefore warranted to test this effect for longer period of time for validation and during its application in the field conditions. In summary, the findings of our study not only help to explain the mechanism involved in mosquito-repellent activity, they may also provide a technical basis for the development of an effective mosquito repellent through a chemical ecology approach.

## Supplementary information

Supplementary Informations.

Supplementary Legends.
