# Supplementary material for: Citronellal perception and transmission by Anopheles gambiae s.s. (Diptera: Culicidae) females
Source: Sci Rep. 2020 Oct 29;10:18615. doi: 10.1038/s41598-020-75782-3 (PMC7596511; doi:10.1038/s41598-020-75782-3)
Supplement: Supplementary file 1 — Supplementary Informations. [file 41598_2020_75782_MOESM1_ESM.pdf]

Citronellal perception and transmission by *Anopheles gambiae* s.s. (Diptera: Culicidae) females

Weijian Wu1[†], Shanshan Li[1†], Min Yang[1], Yongwen Lin[1*], Kaibin Zheng[1], Komivi Senyo Akutse[2]

Table S1 Primers used for amplification of AgamOBPs and AgamOR genes in *Anopheles gambiae* s.s.

Proteins	Reference sequence	Primers	
		F	R
AgamOBP4	NT_078267.5	5' GGAGCAGTAAGTATTTTCACA 3'	5' TTCACCCTTCTTCGTCAT 3'
AgamOBP5	NT_078268.4	5' CGCCTTCTTCTTCATTCC 3'	5' TCAAGCCGCTCACTCATA 3'
AgamOBP20	NT_078265.2	5' ACAGTGAGGAAAAGCGATAA 3'	5' GCACGACAAAAGGGTAAA 3'
AgamORC7	NT_078266.2	5' GCCAGAAGGCGATGACTA 3'	5' TGACAACGGCTCCAAGAA 3'

Table S2 Index of reverse molecular docking prediction for citronellal aim at odorant-binding protein in *Anopheles gambiae s.s*

Pharma Model	Num Feature	Fit	Norm Fit	Num Hydrophobic	Name	Class	Uniplot	Function
1gt5_B_cavity_1	3	2.066	0.6888	3	Odorant-binding protein	NONE	P07435	Involved in chemical odorant binding
3b6x_B_cavity_1	5	2.042	0.4084	5	General odorant-binding protein lush	NONE	O02372	Involved in odorant binding

Table S3: Value of evaluation for the predicted 3D structure of odorant-binding protein and odorant receptor protein in *Anopheles gambiae s.s*

Proteins	Relested stucture	Predited structure			
		Template PDB Code	Sequence Identity	E-Value	GA341
AgamOBP4	3q8i in Protein Data Bank in Europe				
AgamOBP5		1oohA	46.00%	4.00E-23	1
AgamOBP6		1oohA	30.00%	6.00E-23	1
AgamOBP19		1oohA	30.00%	2.00E-19	1
AgamOBP20	3v2l in Protein Data Bank in Europe				
AgamOBP83		1oohA	30.00%	2.00E-18	1
AgamORc7		6c70.1.A	66.67%	3.00E-21	1

Table S4 Auto docking indext of citrolellal with AgamOBPs by using SYBYL-X 2.0

Proteins	Total_Score	Crash	Polar	D_SCORE[a]	PMF_SCORE	G_SCORE	CHEMSCORE	CSCORE[b]
AgamOBP4	5.9289	-0.9325	1.9278	-80.402	-17.4579	-132.6045	-26.4761	2
AgamOBP5	5.8606	-0.4014	0.9778	-67.8984	-5.6024	-134.18	-22.0807	1
AgamOBP20	4.8952	-1.0051	0.82	-76.365	0.4566	-148.7141	-23.7966	3
AgamORC7	5.2105	-0.975	1.4312	-71.7951	-4.4479	-133.3646	-18.2899	5

[a]The score shows the values of the predicted binding energies (kcal/mol).

[b]Concensus Score, was the evaluation for PMF, G and Chem score.

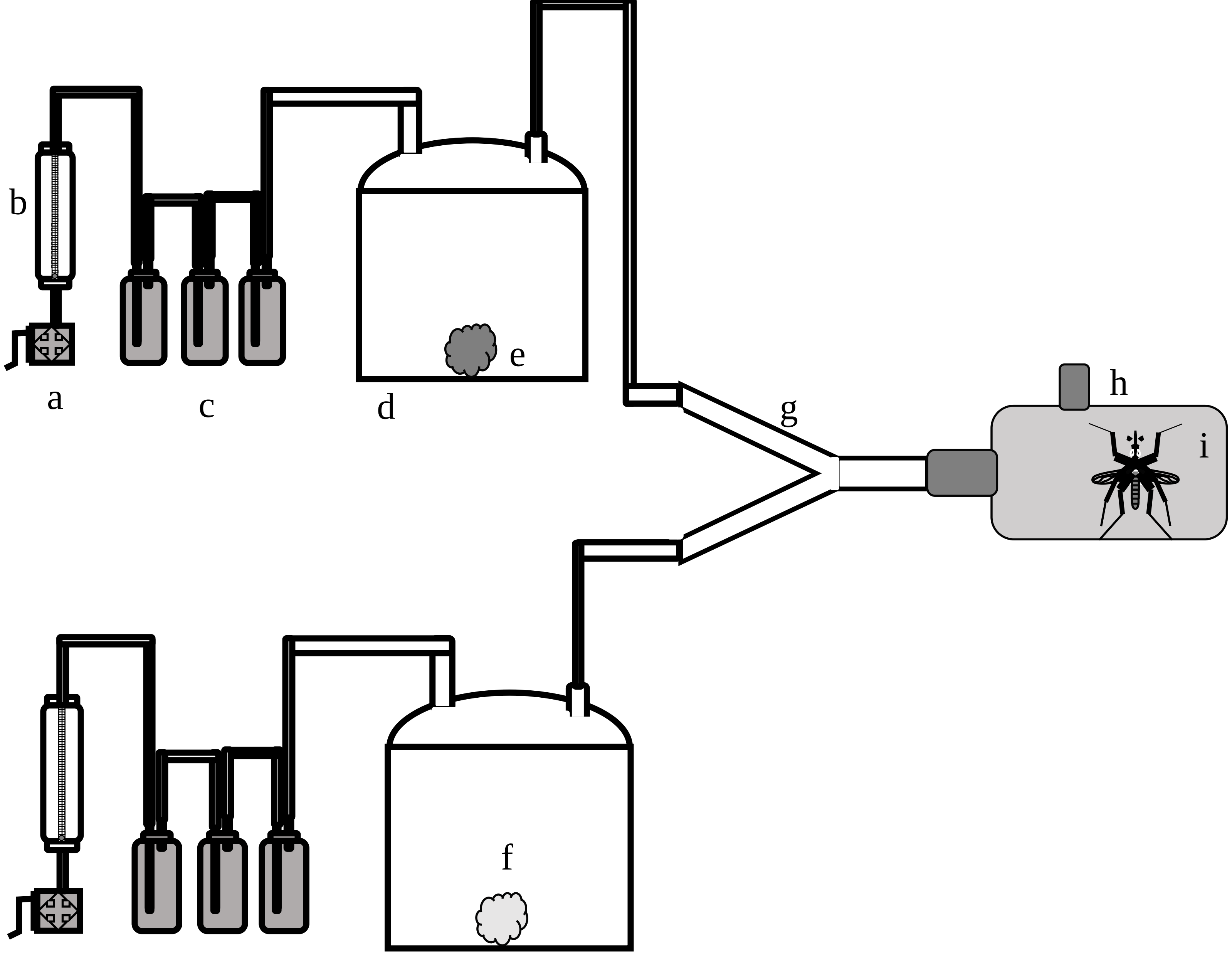

Figure S1

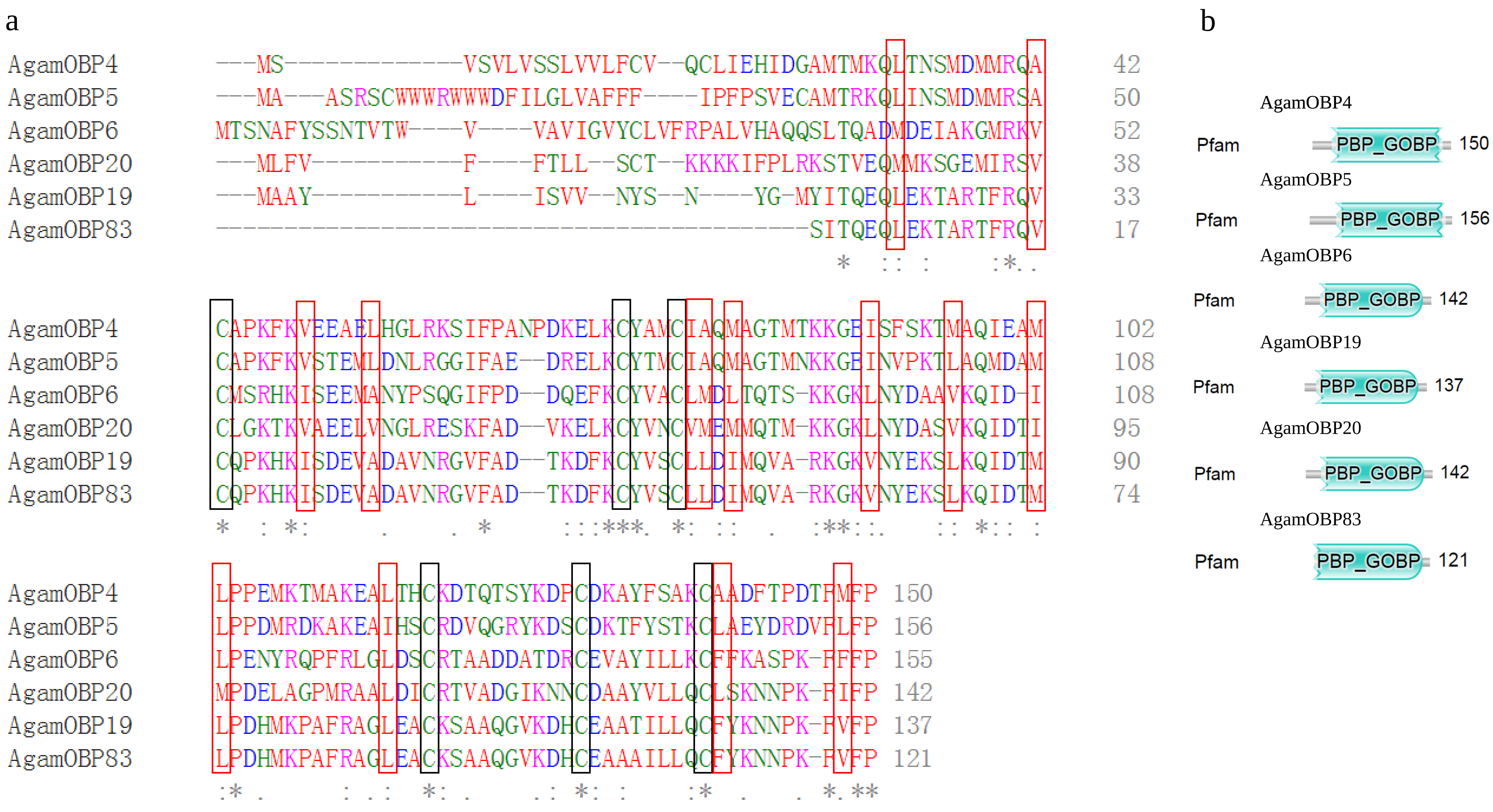
a
b
AgamOBP4
AgamOBP5
AgamOBP6
AgamOBP20
AgamOBP19
AgamOBP83
Pfam
PBP_GOBP

Figure S2

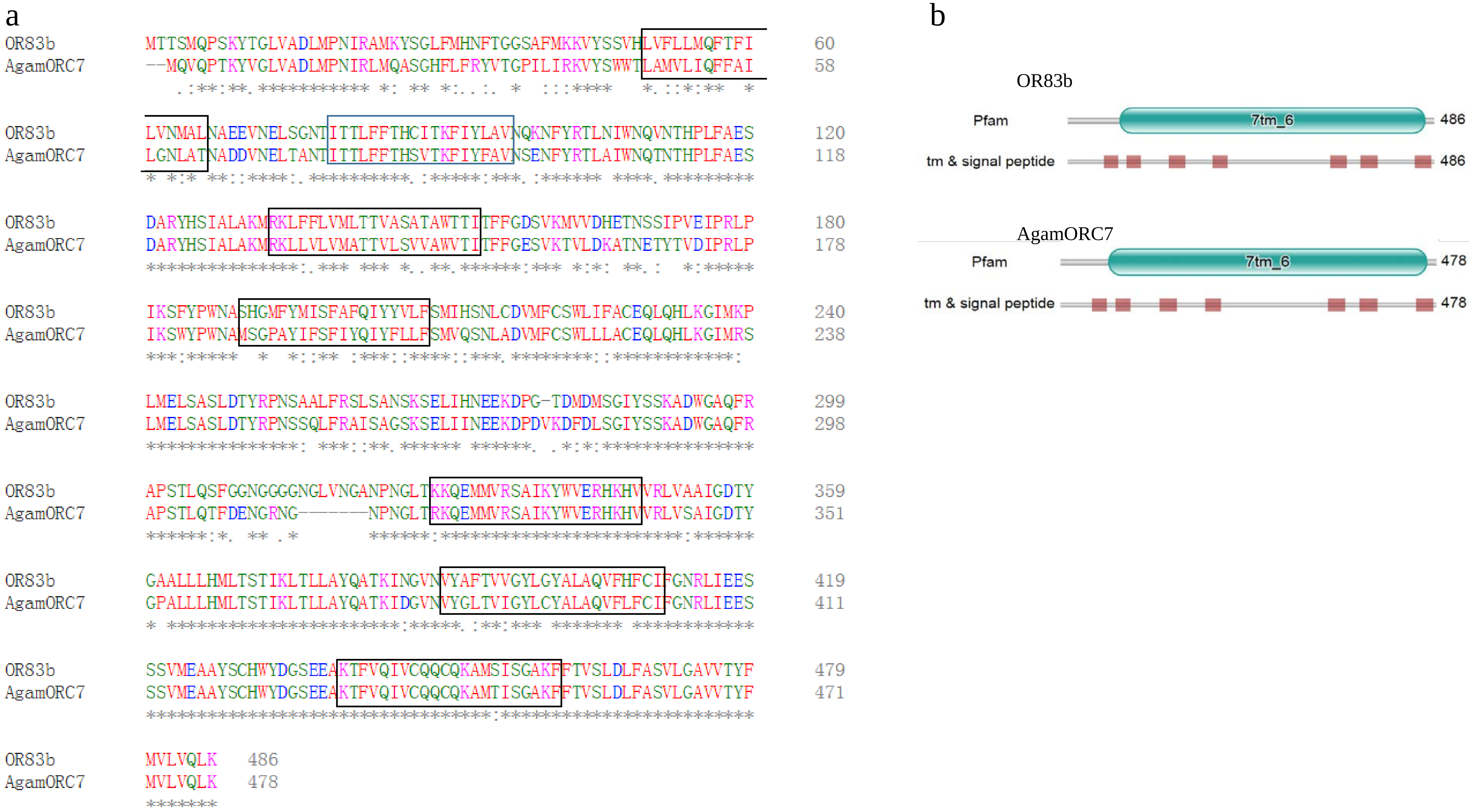
a
OR83b MTTSMQPSKYTGLVADLMPNIRAMKYSGLFMHNFTGGSAFMKKVYSSVHLVFLLMQFTFI 60
AgamORC7 --MQVQPTKYVGLVADLMPNIRLMQASGHFLFRYVTGPILIRKVYSWWTLAMVLIQFFAI 58
OR83b LVNMALNAEEVNELSGNTITTLFFTHCITKFIYLAVNQKNFYRTLNIWNQVNTHPLFAES 120
AgamORC7 LGNLATNADDVNELTANTITTLFFTHSVTKFIYFAVNSENFYRTLAIWNQTNTHPLFAES 118
OR83b DARYHSIALAKMRKLFFLVMLTTVASATAWTTITFFGDSVKMVVDHETNSSIPVEIPRLP 180
AgamORC7 DARYHSIALAKMRKLLVLVMATTVLSVVAWVTITFFGESVKTVLDKATNETYTVDIPRLP 178
OR83b IKSFYPWNASHGMFYMISFAFQIYYVLFSMIHSNLCDVMFCSWLIFACEQLQHLKGIMKP 240
AgamORC7 IKSWYPWNAMSGPAYIFSFIYQIYFLLFSMVQSNLADVMFCSWLLLACEQLQHLKGIMRS 238
OR83b LMELSASLDTYRPNSAALFRSLSANSKSELIHNEEKDPG-TDMDMSGIYSSKADWGAQFR 299
AgamORC7 LMELSASLDTYRPNSSQLFRAISAGSKSELIINEEKDPDVKDFDLSGIYSSKADWGAQFR 298
OR83b APSTLQSFGGNGGGGNGLVNGANPNGLTKKQEMMVRSAIKYWVERHKHVVRLVAAIGDTY 359
AgamORC7 APSTLQTFDENGRNG-------NPNGLTRKQEMMVRSAIKYWVERHKHVVRLVSAIGDTY 351
OR83b GAALLLHMLTSTIKLTLLAYQATKINGVNVYAFTVVGYLGYALAQVFHFCIFGNRLIEES 419
AgamORC7 GPALLLHMLTSTIKLTLLAYQATKIDGVNVYGLTVIGYLCYALAQVFLFCIFGNRLIEES 411
OR83b SSVMEAAYSCHWYDGSEEAKTFVQIVCQQCQKAMSISGAKFFTVSLDLFASVLGAVVTYF 479
AgamORC7 SSVMEAAYSCHWYDGSEEAKTFVQIVCQQCQKAMTISGAKFFTVSLDLFASVLGAVVTYF 471
OR83b MVLVQLK 486
AgamORC7 MVLVQLK 478
b
OR83b
Pfam
7tm_6
486
tm & signal peptide
486
AgamORC7
Pfam
7tm_6
478
tm & signal peptide
478

Figure S3

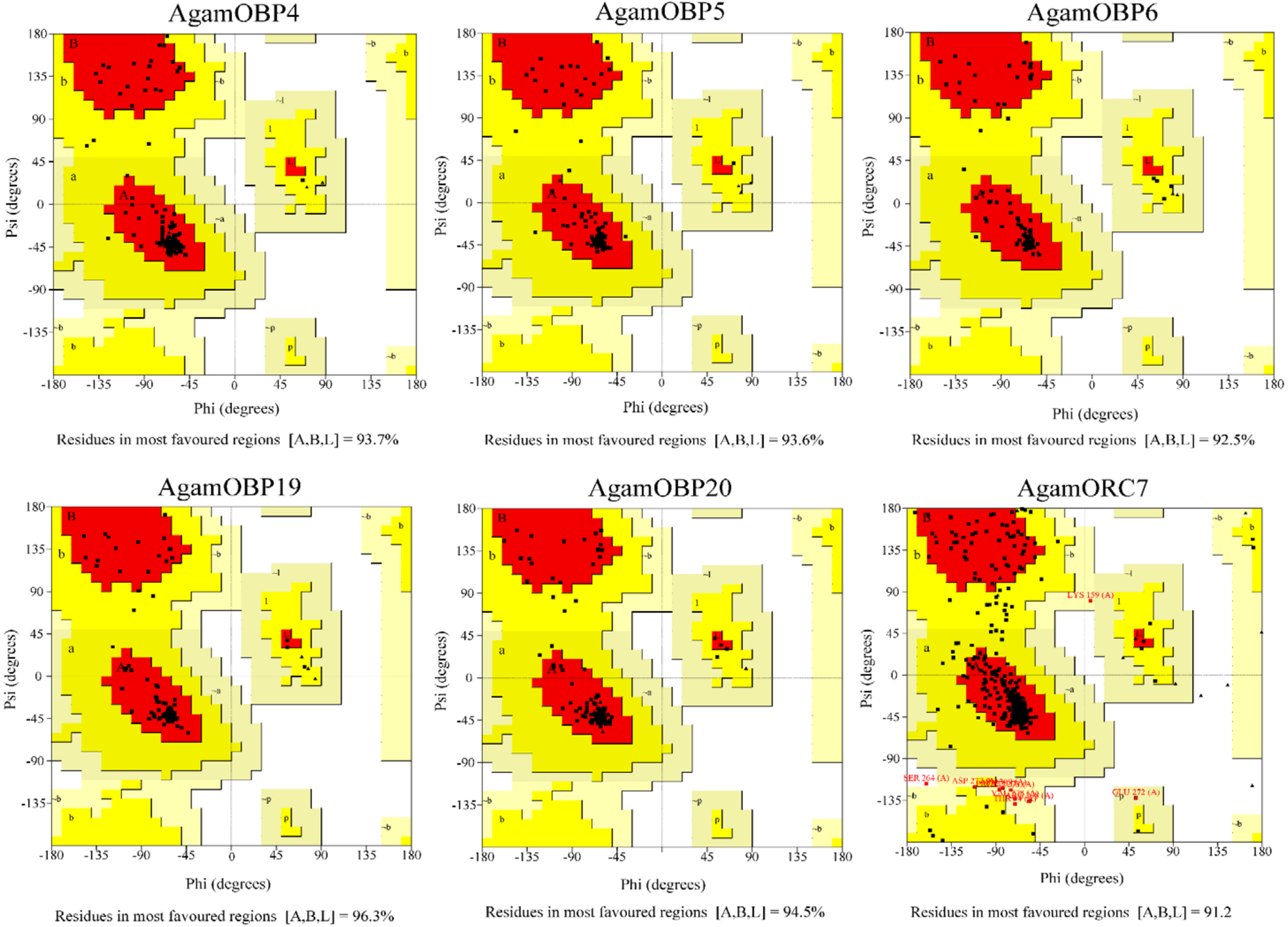
AgamOBP4
Psi (degrees)
Phi (degrees)
Residues in most favoured regions [A,B,L] = 93.7%
AgamOBP5
Psi (degrees)
Phi (degrees)
Residues in most favoured regions [A,B,L] = 93.6%
AgamOBP6
Psi (degrees)
Phi (degrees)
Residues in most favoured regions [A,B,L] = 92.5%
AgamOBP19
Psi (degrees)
Phi (degrees)
Residues in most favoured regions [A,B,L] = 96.3%
AgamOBP20
Psi (degrees)
Phi (degrees)
Residues in most favoured regions [A,B,L] = 94.5%
AgamORC7
LYS 159 (A)
SER 264 (A)
GLU 272 (A)
Psi (degrees)
Phi (degrees)
Residues in most favoured regions [A,B,L] = 91.2

Figure S4

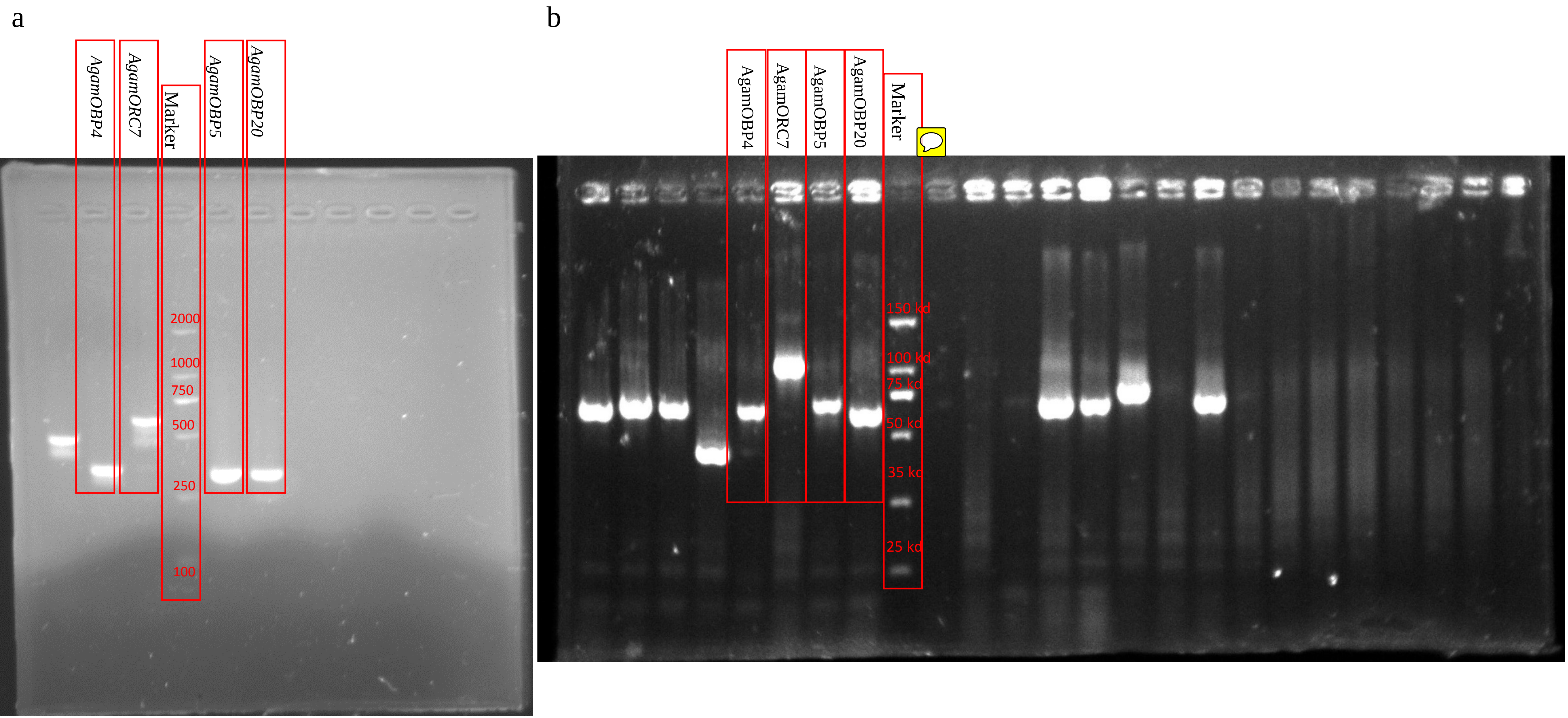
a
AgamOBP4
AgamORC7
Marker
AgamOBP5
AgamOBP20
2000
1000
750
500
250
100
b
AgamOBP4
AgamORC7
AgamOBP5
AgamOBP20
Marker
150 kd
100 kd
75 kd
50 kd
35 kd
25 kd

Figure S5
