# Supplementary material for: Citronellal perception and transmission by Anopheles gambiae s.s. (Diptera: Culicidae) females
Source: Sci Rep. 2020 Oct 29;10:18615. doi: 10.1038/s41598-020-75782-3 (PMC7596511; doi:10.1038/s41598-020-75782-3)
Supplement: Supplementary file 2 — Supplementary Legends. [file 41598_2020_75782_MOESM2_ESM.docx]

# List of supplementary files

**Table S1.** Primers used for amplification of AgamOBPs and AgamOR genes in *Anopheles gambiae s.s*.

**Table S2.** Index of reverse molecular docking prediction for citronellal aim at odorant-binding protein in *A. gambiae*.

**Table S3.** Evaluation value for predicted 3D structure of odorant-binding protein and odorant receptor protein in *A. gambiae*.

**Table S4.** AutoDock index of citrolellal with AgamOBPs by using SYBYL-X 2.0.

**Fig. S1.** Sketch map of Y-tube olfactory meter. a, pump; b, flow meter; c, air purification bottle; d, glass collecting jar; e, cotton ball with citronellal; f, cotton ball without citronellal; g, y-tube; h, air collection bottle; i, mosquito.

**Fig. S2.** Sequences alignment for AgamOBPs with reference OBP protein (a), and Pfam analysis for AgamOBPs (b).

**Fig. S3.** Sequences alignment for AgamOR with reference OR protein (a), and Pfam and transmembrane analysis for AgamORs (b).

**Fig.** **S4.** Ramachandran plot evaluating the 3D structures. Red region, general permitted area; yellow region, theoretical permitted area; gray region, not permitted area.

**Fig. S5.** Gel electrophoresis map of genes (a) and proteins (b).

There are 4 supplementary tables and 5 supplementary figures in the manuscript.
